# Knockdown lncRNA DLEU1 Inhibits Gliomas Progression and Promotes Temozolomide Chemosensitivity by Regulating Autophagy

**DOI:** 10.3389/fphar.2020.560543

**Published:** 2020-12-09

**Authors:** Qiao-Li Lv, Li-Chong Wang, Dang-Chi Li, Qian-Xia Lin, Xiao-Li Shen, Hai-Yun Liu, Min Li, Yu-Long Ji, Chong-Zhen Qin, Shu-Hui Chen

**Affiliations:** ^1^ Jiangxi Key Laboratory of Translational Cancer Research, Department of Head and Neck Surgery, Jiangxi Cancer Hospital of Nanchang University, Nanchang, China; ^2^ Department of Neurosurgery, The Second Affiliated Hospital of Nanchang University, Nanchang, China; ^3^ Jiangxi University of Technology High School, Nanchang, China; ^4^ Jiangxi University of Traditional Chinese Medicine, Nanchang, China; ^5^ Department of Head and Neck Surgery, Jiangxi Cancer Hospital, Nanchang, China; ^6^ Department of Pharmacy, The First Affiliated Hospital of Zhengzhou University, Zhengzhou, China; ^7^ Department of Radiation Oncology, Jiangxi Cancer Hospital of Nanchang University, Nanchang, China

**Keywords:** LncRNA DLEU1, Glioma, Temozolomide, Autophagy, Epithelial-Mesenchymal Transition

## Abstract

Gliomas are the most fatal malignant cerebral tumors. Temozolomide (TMZ), as the primary chemotherapy drug, has been widely used in clinics. However, resistance of TMZ still remains to poor defined. LncRNAs have been reported to play crucial roles in progression of various cancers and resistance of multiple drugs. However, the biological function and underlying mechanisms of most lncRNAs in glioma still remains unclear. Based on the TCGA database, a total of 94 differentially expressed lncRNAs, including 16 up-regulated genes and 78 downregulated genes were identified between gliomas and normal brain tissues. Subsequently, lncRNA DLEU1, HOTAIR, and LOC00132111 were tested to be significantly related to overall survival (OS) between high- and low-expression groups. Additionally, we verified that lncRNA DLEU1 was high expressed in 108 gliomas, compared with 19 normal brain tissues. And high expression of lncRNA DLEU1 predicted a poor prognosis (HR = 1.703, 95%CI: 1.133–2.917, *p*-value = 0.0159). Moreover, functional assays revealed that knockdown of lncRNA DLEU1 could suppress the proliferation by inducing cell cycle arrest at G1 phase and reducing the S phase by down-regulating the CyclinD1 and *p*-AKT, as the well as migration and invasion by inhibiting the epithelial–mesenchymal transition (EMT) markers, such as ZEB1, N-cadherin, β-catenin and snail in glioma cells. Furthermore, silencing lncRNA DLEU1 suppressed TMZ-activated autophagy via regulating the expression of P62 and LC3, and promoted sensitivity of glioma cells to TMZ by triggering apoptosis. Conclusively, our study indicated that lncRNA DLEU1 might perform as a prognostic potential target and underlying therapeutic target for sensitivity of glioma to TMZ.

## Introduction

Glioma, accounting for approximately 80% of malignant tumors, is one of the most primary and fatal intracranial tumors (Jin et al., 2019; Wu et al., 2019). Despite advances in diagnosis and therapy such as surgical resection followed by adjuvant radiotherapy and chemotherapy, patients with glioblastoma have a median survival time of merely 12–15 months Parsons et al., 2008; Mostafa et al., 2016). Temozolomide (TMZ), the primary chemotherapeutic drug for gliomas, was reported to prolong the survival time of glioblastoma patients (Nanegrungsunk et al., 2015; Wait et al., 2015). Nevertheless, TMZ resistance mechanisms were still ubiquitous. Although several biomarkers such as MGMT, STAT3, and APNG have been explored to be associated with the sensitivity of TMZ (Jacinto and Esteller, 2007; Agnihotri et al., 2012; Lee et al., 2011), TMZ resistance in gliomas is still incompletely elaborated. Thus, the research for novel chemotherapeutic targets is crucial for glioma therapy.

Long non-coding RNAs (lncRNAs) were defined as long RNA transcripts (>200 nucleotides) with no protein-coding capability. However, a few articles discovered the peptide-coding ability of certain lncRNAs (Necsulea et al., 2014; Quinn and Chang, 2016). Recently, increasing studies have revealed that lncRNA plays an important and various role in progression of glioma (Lv et al., 2016a; Chen H. et al., 2019; Li C. et al., 2019). For example, lncRNA ZEB1-AS1, which serves as an oncogene, could promote tumorigenesis of gliomas by activating epithelial-to-mesenchymal transition (EMT), such as ZEB1, N-cadherin, and MMP2 protein markers (Lv et al., 2016a). On the contrary, lncRNA CPS1-IT1 was discovered to be an anti-oncogene gene in glioma (Chen H. et al., 2019). Moreover, linc00467 and LncRNA HANR were found to aggravate the malignant progression and promote invasion and proliferation of glioma cells by targeting miRNA-485-5p and miRNA-335, respectively (Jiang and Liu, 2020; Wang et al., 2020). These findings implied that more detailed mechanisms of lncRNA still remained unclear. It is important to illuminate the potential molecular mechanisms and explore prognostic biomarkers to help progress of therapeutic targets and strategies for glioma.

The Cancer Genome Atlas (TCGA), a landmark cancer genomics program, is widely used in the genetic research of cancer. Based on RNA-seq data from TCGA, we demonstrated that the differential expression gene in glioma, named lncRNA deleted in lymphocytic leukemia 1 (lncRNA DLEU1), was dramatically associated with a poor prognosis. Emerging research has reported that lncRNA DLEU1 performed an indispensable role in the genesis and progression of various tumors such as gastric cancer, osteosarcoma, pancreatic ductal adenocarcinoma, non-small cell lung cancer, and breast cancer, as well as resistance to chemotherapy in tumors (Li X. et al., 2018; Chen X. et al., 2019; Gao et al., 2019; Zhang et al., 2019; Wang C. et al., 2019; Li Y. et al., 2019). However, the progression and TMZ chemosensitivity of lncRNA DLEU1 in glioma are still vague.

In the current study, we investigated the differential expression of lncRNA DLEU1 between 108 gliomas and 19 normal cerebral trauma tissues as well as the prognostic value. In addition, we examined the potential molecular mechanisms of lncRNA DLEU1 in the regulation of proliferation, migration, and invasion in U87 and U251 cells. Furthermore, we revealed the underlying mechanism of lncRN ADLEU1 involved in sensitivity of glioma cells to TMZ, which provided a potential target for the individualized therapy of temozolomide in glioma.

## Materials and Methods

### Patients Samples and Follow-Up

A total of 108 human glioma tissues and clinical information were acquired from patients who underwent surgical resection before treatment with radiation and chemotherapy between November 2010 and June 2013 in the Department of Neurosurgery, Xiangya Hospital (Hunan, China). Nineteen normal brain specimens obtained from patients with cerebral trauma surgery were normalized as controls. Informed consents were provided to all patients or their family members. All participants signed the written informed contents. This study was approved by the Hospital Ethics Committee (No. 201803806).

### Microarray Analysis

Raw RNA-seq data (count files) and clinical information were obtained from the TCGA database (https://tcga-data.nci.nih.gov/tcga/). Then the expression value of lncRNA was flited from the raw files. The differential expressed genes were identified by DEseq with a threshold: adjusted-*p* < 0.05, log2FoldChange > 1. All *p*-values were adjusted by the Benjamini–Hochberg’s method. Then the association between the expression of differentially expressed lncRNAs and overall survival was evaluated by univariate Cox proportional hazards regression analysis using the survival R package of R 3.6.1 (https://www.r-project.org/). LncRNAs with *p*-value<0.05 were considered as candidate variables. The log-rank test was performed to evaluate the prognostic value of lncRNAs.

### Functional Enrichment Analysis

To identify potential biological processes and pathways that the filtrated lncRNAs were involved in, functional enrichment analysis was performed. First, we integrated differential lncRNAs’ neighboring (10 Kb) mRNAs as underlying target genes (TGs). Differentially expressed lncRNAs and mRNA sequences were extracted and primed by blast (e < 1E-5), and then screened again by the software [RNAplex (G < −20)] to identify the possible target genes of lncRNA. Kyoto Encyclopedia of Genes and Genomes (KEGG) pathway enrichment analysis was carried out for those target genes using the database (http://www.genome.jp/). Gene ontology (GO) was confirmed by hypergeometric distribution. The *p*-value <0.05 was set as the cutoff criterion for both GO and KEGG functional analysis.

### Cell Lines and Materials

The human glioma cell lines (U87 and U251) were acquired from the American Type Culture Collection (ATCC) and kept in Dulbecco’s modified Eagle’s medium (DMEM, HyClone, United States) supplemented with 10% fetal bovine serum (FBS, GIBCO, Carlsbad, California, United States) and in a 5% CO2 humidified incubator at 37°C. TMZ was purchased from Sigma- Aldrich (St. Louis, Missouri, United States).

### Cell Transfection

Two different small interfering RNA against lncRNA DLEU1 (siRNA-DLEU1) and negative control siRNA (NC) were synthesized by RiboBio (Guangzhou, China). Cells that were growing exponentially were seeded in six- well plates and cultured overnight. When the plated cells arrived at approximately 50–60% confluence, the siRNAs were transiently transfected into U87 and U251 cells using Lipofectamine® RNAiMAX Transfection Reagent (Invitrogen, Carlsbad, California, United States) according to the manufacturer’s directions.

### RNA Extraction and Real-Time Polymerase Chain Reaction

Total RNA was isolated from cultured cells or tissues samples using TRIzol reagent (Invitrogen, Carlsbad, California, United States). The cDNA was generated by reverse transcription of 1 µg of total RNA using Thermo Scientific Revert Aid First Strand cDNA Synthesis Kit (Thermo Fisher Scientific, Wilmington, Delaware, United States). According to the manufacturer’s instructions, real-time PCR was conducted by using the SYBR Green Real-Time PCR Kit (Takara, Dalian, China) on a LightCycler480 (Roche, San Francisco, California, United States) to detect the expression of lncRNA DLEU1, with GAPDH as a normalizing control. PCR was performed at the following conditions: 95.0°C for 3 min, and 40 circles of 95.0°C for 10 s and 60°C for 30 s. The relative quantitative value was evaluated by the 2^−ΔΔCT^ method. The primers for lncRNA DLEU1 were F: 5′-GCG​GAG​GTG​AAG​TGA​ACT​TAG​A-3′; and R: 5′-CTC​CTA​AGC​AGG​ACC​CGT​ATT-3′. The primers for GAPDH were F: 5′-CCC​ATC​ACC​ATC​TTC​CAG​GAG-3′; and R: 5′-GTT​GTC​ATG​GAT​GAC​CTT​GGC-3′.

### Western Blotting Analysis

The proteins were extracted and measured from cultured cells as previously described (Lv et al., 2016b). Anti-AKT, anti-*p*-AKT, anti-N-cadherin, anti-Snail, anti-P62, anti-LC3, anti-GAPDH and anti-β-actin antibodies were purchased from CST Biotechnology (Boston, Massachusetts, United States). The relative protein expression was analyzed based on gray value. Each test was repeated three times. The β-actin and GAPDH were used as the internal references.

### Cell Proliferation Assay

The effect of siRNA-DLEU1 on cell proliferation was observed by using MTS assay (Cell Titer 96 aqueous one solution reagent, Promega, Madison, Wisconsin, United States). At 24 h before the experiment, cells were plated in 96‐well plates at a density of 3,000 cells in 100 μl medium per well. Then, the cells were divided into three groups that were transfected with NC, siRNA1-DLEU1, or siRNA2-DLEU1 and cultured for 24, 48, 72, and 96 h. The cell viability was assessed by MTS 10 µl of MTS diluted in 90 µl of culture medium, and then added the medium into each well and incubated at 37°C for 30 min. The absorbance at 490 nm was detected on a microplate reader (Bio-Rad Laboratories, Inc., Hercules, California, United States). All experiments were repeated three times.

### Colony Formation Assay

U87 and U251 cells were seeded in six-well plates with the density of 600 cells per well. Then cells were transfected with NC, siRNA1-DLEU1, or siRNA2-DLEU1. After incubating in a 5% CO_2_ humidified incubator at 37°C for approximately 15 days, the cells were fixed with 4% paraformaldehyde for 15 min, and stained with 0.1% crystal violet (Beyotime Institute of Biotechnology, Shanghai, China) for 20 min. The stained colonies were photographed using a high-resolution camera. The number of colonies was counted by the microscope.

### Wound Healing Assay

U87 and U251 cells were seeded in six-well plates for overnight and transfected with siRNA1-DLEU1, siRNA2-DLEU1, and NC. When the number of cells reached approximately 80–90% density, we made a wound across the center of the well using a 1 ml plastic pipette tip. Then the cells were softly washed with phosphate-buffered saline (PBS) three times and cultured in a medium containing 2% FBS at 37°C. After wounding, the size of the wound was photographed by microscope at 0 and 24 h. Three separate experiments were repeated three times.

### Transwell Invasion Assay

The cell invasion assay was performed using 24-well transwell chambers (Corning, Tewksbury, Massachusetts, United States) coated with Matrigel. Both transfected and non-transfected U87 and U251 cell harvested and resuspended in serum-free DMEM medium were seeded into the upper chambers at a density of 1.0 × 10^5^ cells/mL in 0.2 ml of 1% FBS medium, while medium containing 10% FBS was seeded into lower chambers. After incubation for 24 h at 37°C, the invasive or migrative cells were fixed with 4% formalin for 10 min and stained with crystal violet for 10 min. After being washed softly with PBS three times, cells were counted by a light microscope in randomly selected fields.

### Hoechst Staining

U87 and U251 cells were seeded into six-well plates, and transfected with siRNA1-DLEU1, siRNA2-DLEU1, or NC. Briefly, after incubation for 48 h, cells were fixed by 4% (v/v) formaldehyde for 10 min and washed twice with PBS, then stained with Hoechst 33,342 for 15 min and washed softly three times with PBS. The apoptotic cells were observed and photographed under fluorescence microscope.

### Flow Cytometry Analysis

For cell cycle analysis, transfected U87 and U251 cells were plated into six-well plates and cultured in an incubator at 37°C with 5% CO2 for 48 h. All cells were harvested by trypsinization and fixed with precooled 70% ethanol at −20°C overnight. After being centrifuged at 1,000 g for 5 min, the cells were then resuspended and incubated in 300 μl staining buffer, 10 μl propidium iodide (PI), and 5 uL rnase (Beyotime Institute of Biotechnology, Shanghai, China) for 20 min at 37°C for in the dark. Cells were analyzed by FC500 flow cytometry (Beckman Coulter, Bethesda, Massachusetts, United States). The cell cycle distribution in the G0/G1, S, and G2/M phases was calculated with the FlowJo software (version 7.6.5; Tree Star, Ashland, United States).

Cell apoptosis assay, according to manufacturer’s instructions, was evaluated by Annevin V-FITC/PI (Beyotime Institute of Biotechnology, Shanghai, China). Briefly, U87 and U251 cells transfected with siRNA1-DLEU1, siRNA2-DLEU1 or NC were collected by trypsin without EDTA and centrifuged at 1,000 g for 5 min. The cells were resuspended with staining solution which contained 195 μl binding buffer, 5 μl Annexin V-FITC, and 10 μl PI, then incubated at 37°C for 20 min in the dark. Then the cells were immediately analyzed by flow cytometry (Beckman Coulter, Atlanta, Georgia, United States).

### Analysis of Autophagic Flux

According to the manufacturer’s instructions, the U87 and U251 cells were transfected with a tandem RFP-GFP–tagged LC-3. The transfected cells were starved for 16 h and changed the complete medium. Then these cells were transfected by siRNA-DLUE1 and treated with TMZ at 300 μM for 48 h. Next, these cells were fixed with 4% paraformaldehyde for 10 min and washed for three times with PBS. Finally, the GFP/RFP images were observed with a laser scanning confocal microscope.

### Statistical Analysis

All the experimental data were analyzed by SPSS software (version 20.0). All experiments were separately repeated three times. Student’s t-test was used to analyze the relationship between siRNA-DLEU1 and NC groups. The Kaplan-Meier method and a log-rank test were performed for comparisons of survival rate. Data were presented as plus–minus (±) standard deviation (SD). *p* <0.05 was considered statistically significant.

## Results

### Differentially Expressed lncRNAs Between Gliomas and Normal Brain Tissues

**FIGURE 1:**
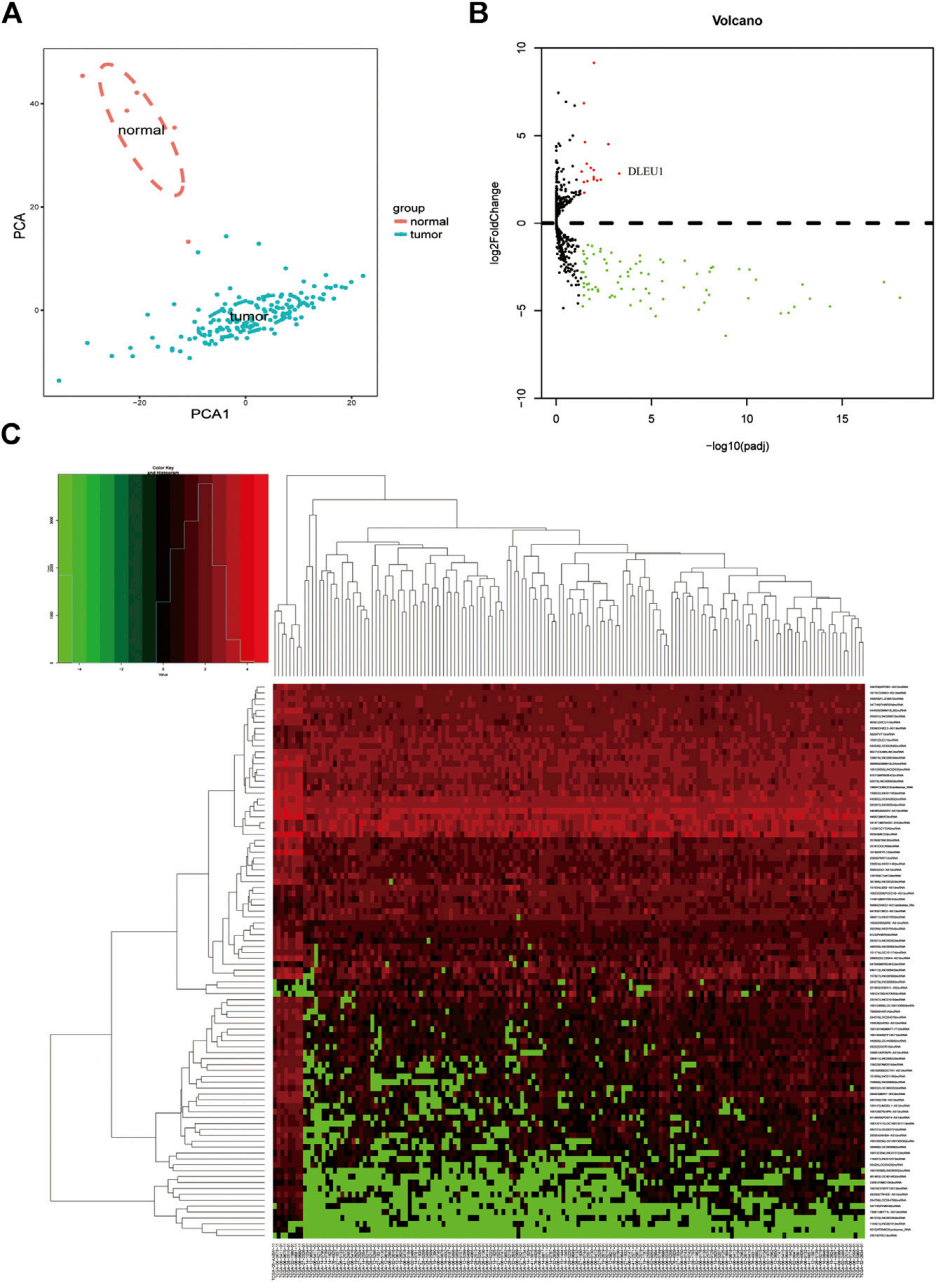
Differentially expressed lncRNAs between gliomas and normal tissues. **(A)** PCA of the total RNA expression profile in TCGA, normal brain tissues were marked with red. **(B)** Volcano diagram showed the DGEs in TCGA database. **(C)** The cluster analysis of the differentially expressed lncRNAs.

Based on the analysis of the TCGA database, we compared the different expressions of lncRNAs between glioma (*n* = 153) and normal brain tissues (*n* = 5). Principal component analysis (PCA) was calculated to compare the transcriptional profiles, showing that there was a clear distinction between the two groups (Figure 1A). According to the cutoff criteria (adjusted *p* <0.05, log2FoldChange > 1), a total of 94 differentially expressed lncRNAs, including 16 upregulated genes and 78 downregulated genes were identified between gliomas and normal tissues (Figure 1B). The cluster analysis showed that the glioma samples could be clearly distinguished from the normal controls with the expression of differentially expressed lncRNAs (Figure 1C).

### Establishment of the lncRNA Associated With Overall Survival of Glioma Patients

**FIGURE 2:**
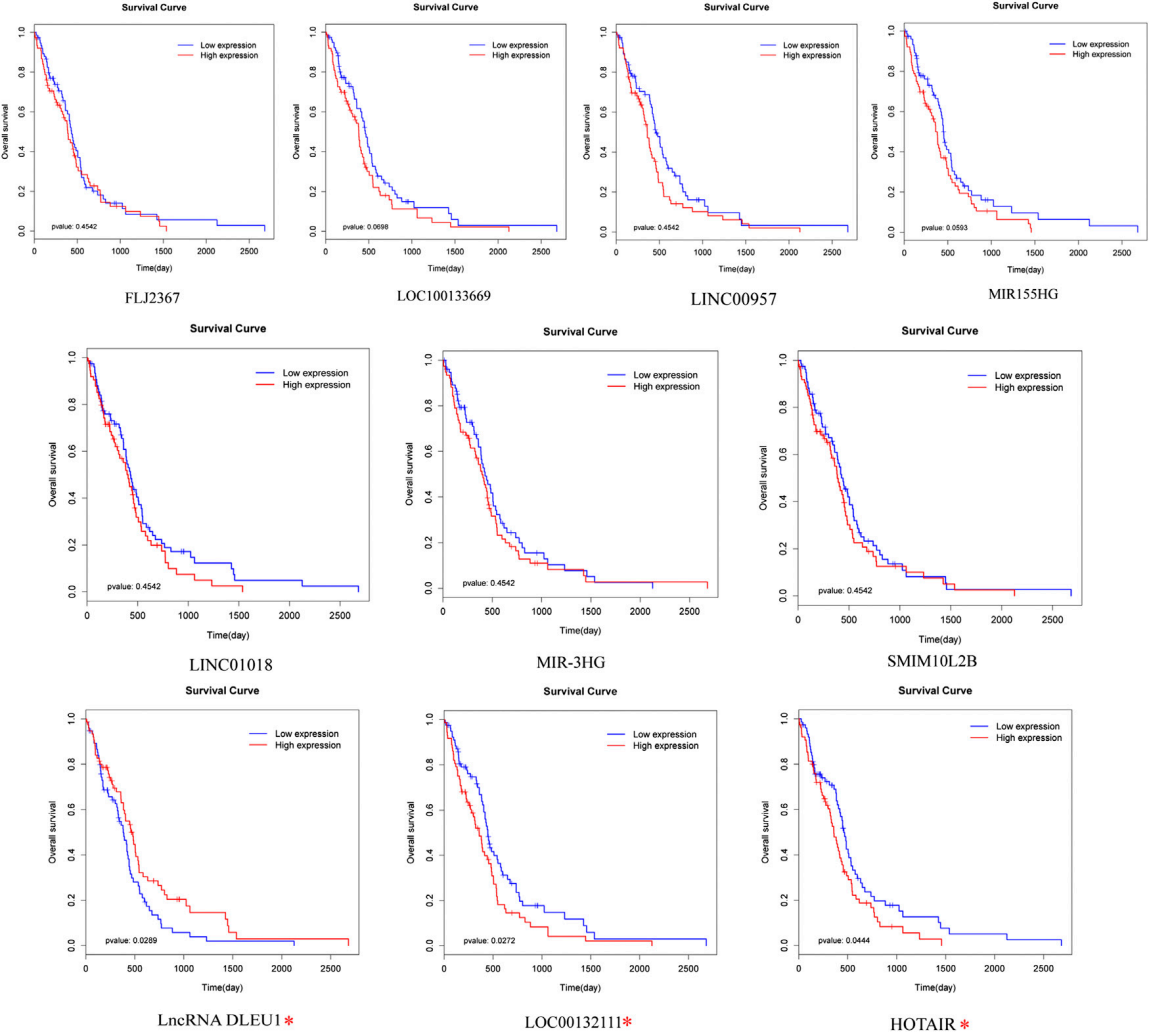
Prognostic evaluation of the 10 lncRNAs in glioma patients by log-rank test. LncRNA DLEU1, HOTAIR, and LOC00132111 were significantly related to OS between high and low expression groups. *p < 0.05.

To identify prognosis-related lncRNAs, univariate Cox regression analysis was determine to evaluate the associations between the expression level of 94 differentially expressed lncRNAs and patients’ overall survival. The result showed that 10 lncRNAs were remarkably associated to overall survival (Table 1, *p* <0.05). Patients were divided into high and low expression groups by using the median expression of lncRNA as a cutoff point and the significance was determined between the two groups using a log-rank test with *p* < 0.05. The log-rank test was performed showing that only lncRNA DLEU1, LOC00132111, and HOTAIR were significantly associated with the prognosis between high and low expression groups (Figure 2, *p <0.05).

### Identification of the 10 lncRNA Related Biological Processes and Pathways

**FIGURE 3:**
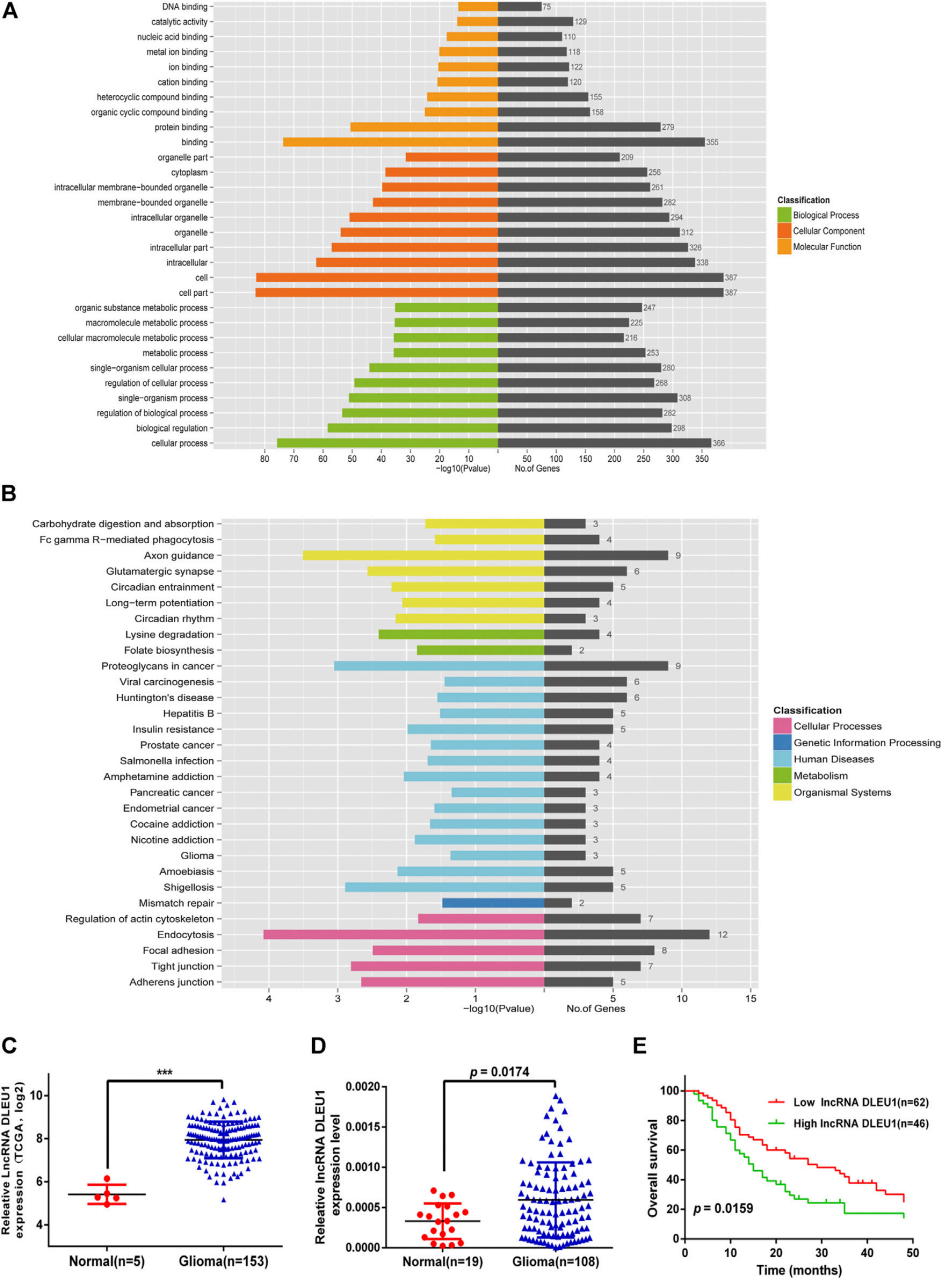
Go and KEGG pathway for lncRNA-related target genes. Expression of lncRNA DLEU1 was upregulated in glioma tissues and predicted poor prognosis. **(A)** Enrichment analysis of top 10 GO BP and MF terms for lncRNA-related TGs. **(B)** KEGG pathway for lncRNA-related TGs. **(C)** The expression of lncRNA DLEU1 was significantly upregulated in gliomas based on TCGA. **(D)** Comparison of lncRNA DLUE1 expression levels between 108 glioblastoma tissues and 19 normal brain tissues. **(E)** Comparison of overall survival (OS) between patients with lncRNA DLEU1 high and low expression levels in 108 patients. ***p < 0.001.

To explore the potential function of 10 prognosis-related lncRNAs, we performed GO and KEGG functional enrichment analysis for the target mRNAs co-expressed with the lncRNAs. As shown in Figure 3A, the target mRNAs were enriched in the top 10 significant terms for each of the following: the biological process (BP), cellular component (CC), and molecular function (MF). In the BP group, genes were mainly enriched in organic cyclic compound binding, binding, and protein binding. In the CC group, genes were enriched in the cell part, intracellular part, and organelle. In the MF group, genes were enriched in cellular process regulation of biological process cellular macromolecule metabolic process. Moreover, Kyoto Encyclopedia of Genes and Genomes (KEGG) pathway analysis was calculated, showing these genes were enriched in mismatch repair, adherens junction, glioma, insulin resistance, and Fc gamma R−mediated phagocytosis (Figure 3B).

### Expression of lncRNA DLEU1 Was Up-Regulated in Glioma Tissues and Predicted Poor Prognosis

Base on the TCGA datasets, as shown in Figure 3C, the expression of lncRNA DLEU1 was significantly up-regulated in gliomas (adjusted *p* = 0.000501). The aforementioned studies had shown that lncRNA DLEU1 predicted a poor prognosis. To further prove our finding based on the TCGA dataset, we collected 127 tissues from Xiangya Hospital and detected the expression level of lncRNA DLEU1 in gliomas (*n* = 108) and normal brain tissues (*n* = 19) by qRT-PCR, discovering the expression feature was similar to our results from TCGA analysis. lncRNA DLEU1 was dramatically high expressed in 108 gliomas compared with 19 the traumatic brain tissues (Figure 3D, *p* = 0.0174). Meanwhile, by using the Kaplan-Meier method, the survival curve also showed that the high expression of lncRNA DLEU1 was associated with a worse prognosis, which was compatible with the previous results (Figure 3E, HR = 1.703, 95%CI:1.133–2.917, *p* = 0.0159).

### Knockdown of lncRNA DLEU1 Suppressed the Proliferation of Glioma Cells

**FIGURE 4:**
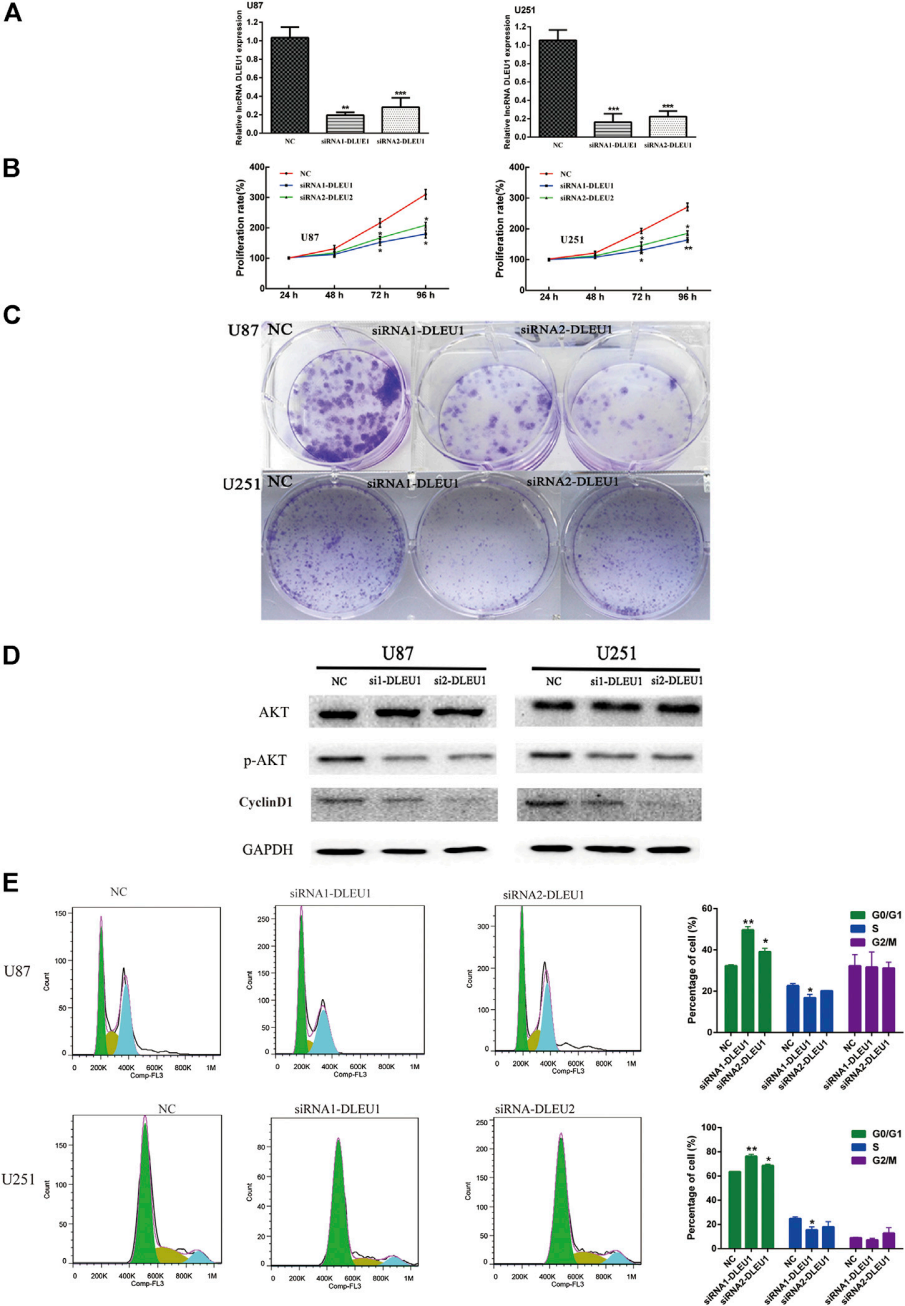
siRNA-DLEU1 inhibited the proliferation of glioma cells via interfering with expression levels of cell cycle–related proteins. **(A)** LncRNA DLEU1 siRNAs interference efficiency was examined by RT-qPCR. **(B)** Knockdown by si-DLEU1 transfection suppressed the proliferation of U87 and U251cells by MTS assay. **(C)** Knockdown of DLEU1 impaired clone formation ability of glioma cells. **(D)** Changes in protein expression of p-AKT and CyclinD1 after transfecting siRNA-DLEU1. **(E)** Changes in proportion of U87 and U251 cells in different phases with lncRNA DLEU1 depletion. Left panel: representative flow cytometry analysis results; right panel: quantitative analysis of the percentage of different phase. (*p < 0.05, **p < 0.01, ***p < 0.001), NS, not significant.

To further investigate whether lncRNA DLEU1 was involved in the progression of glioma, we evaluated the effect of lncRNA DLEU1 on glioma proliferation. First, on account of the high expression of lncRNA DLEU1 in glioma tissues, two specific siRNAs were adopted to silence lncRNA DLEU1 expression. After transfection and incubation for 48 h, the interference efficiency of siRNAs was validated by RT-qPCR in U87 and U251 cells, showing that the siRNA1-DLUE1 and siRNA2-DLEU1 effectively decrease lncRNA DLEU1 expression in glioblastoma cells (Figure 4A, *p <0.05, **p <0.01, ***p <0.001). Then, compared with the NC group, MTS assay revealed that knockdown of lncRNA DLEU1 dramatically depressed the proliferation of U87 and U251 cells shown by proliferation curves (Figure 4B, *p <0.05, **p <0.01). Finally, as shown in Figure 4C, colony-formation assay indicated that the loss of lncRNA DLEU1 remarkably reduced the counts of newly formed colonies in U87 and U251 cells.

### Silenced lncRNA DLUE1 Interfered With Cell Cycle and the Expression Levels of Cell Cycle Related Proteins in Glioma Cells

In order to measure whether the impaired proliferation of glioma cells was affected by knockdown of lncRNA DLEU1, we analyzed the cell cycle distribution of glioma cells after silencing lncRNA DLEU1 by flow cytometry analysis. Efforts in our laboratory implied that knockdown of lncRNA DLEU1 contributed to the accumulation in the G0/G1 phase while decreasing the percentage of S1 phase (Figure 4E, *p <0.05, **p <0.01, ***p <0.001). Meanwhile, western blot analysis was performed to explore the protein expression of G1/S transition markers, showing that knockdown of lncRNA DLEU1 significantly decreased the protein expression of *p*-AKT and Cyclin D1 (Figure 4D). All he aforementioned results indicated that silenced lncRNADLUE1 could depress the proliferation of glioma cells via interfering the cell cycle.

### Knockdown of lncRNA DLEU1 Suppressed Migration and Invasion Abilities of Glioma Cells by Epithelial-Mesenchymal Transition

**FIGURE 5:**
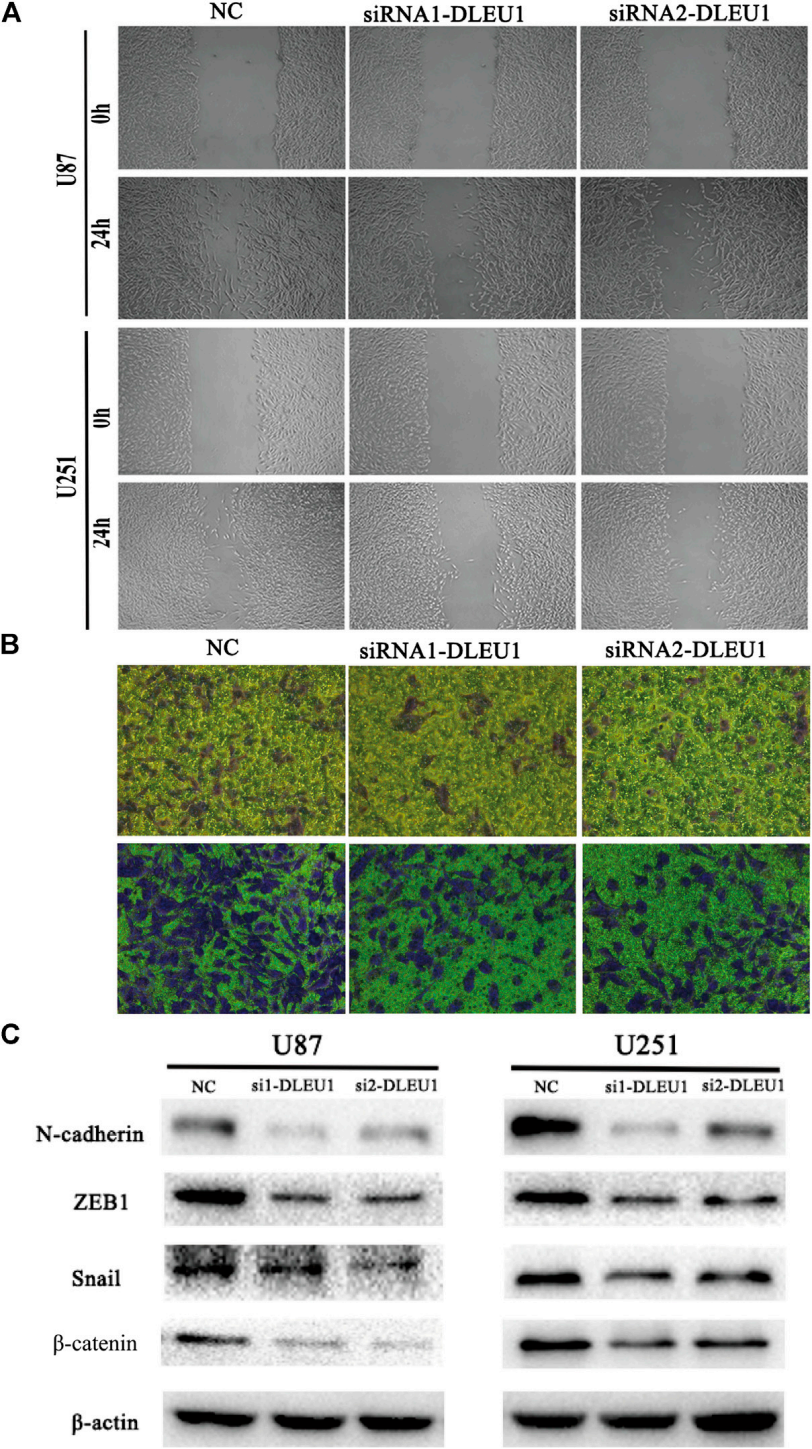
The depletion of lncRNA DLEU1 inhibited the migration and invasion of glioma cells by suppressing EMT. **(A,B)** Wound healing and transwell assays revealed that siRNA1-DLEU1 and siRNA2-DLEU1 could significantly inhibit cell migration and invasion in U87 and U251 cell lines, compared with NC groups. **(C)** The changes of the expressions of EMT markers such as ZEB1, N-cadherin, β-catenin, and snail by silencing lncRNA DLEU1.

To further explore the functions of lncRNA DLEU1 in migration and invasion of glioma cells, as shown in Figure 5A, the wound healing assay were performed, demonstrating that knocking down lncRNA DLEU1 in U87 and U251 cells could significantly inhibit migration in glioma cells, compared with the NC groups after 24 h of incubation. Additionally, we observed that more U87 and U251 cells invaded from the top chambers to the bottom chambers in the NC groups, rather than in the siRNA1-DLEU1 and siRNA2-DLEU1 groups by the transwell invasion assay (Figure 5B).

The preceding results indicated that lncRNA DLEU1 could promote the migration and invasion of glioma cells, which meant lncRNA DLEU1 might be involved in progression of EMT, which plays a significant role in the infiltration and metastasis of tumors. Therefore, western blot analysis was calculated to evaluate the expression of well-known EMT markers, such as ZEB1, β-catenin, N-cadherin, and snail. As shown in Figure 5C, the protein levels of the ZEB1, N-cadherin, β-catenin, and snail were remarkably reduced in siRNA-DLEU1 groups compared with NC groups, indicating that siRNA-DLEU1 could inhibit migration and invasion of glioma cells by suppressing EMT markers.

### Knockdown of lncRNA DLEU1 Contributed to Temozolomide Sensitivity in Glioma Cells by Promoting Apoptosis

**FIGURE 6:**
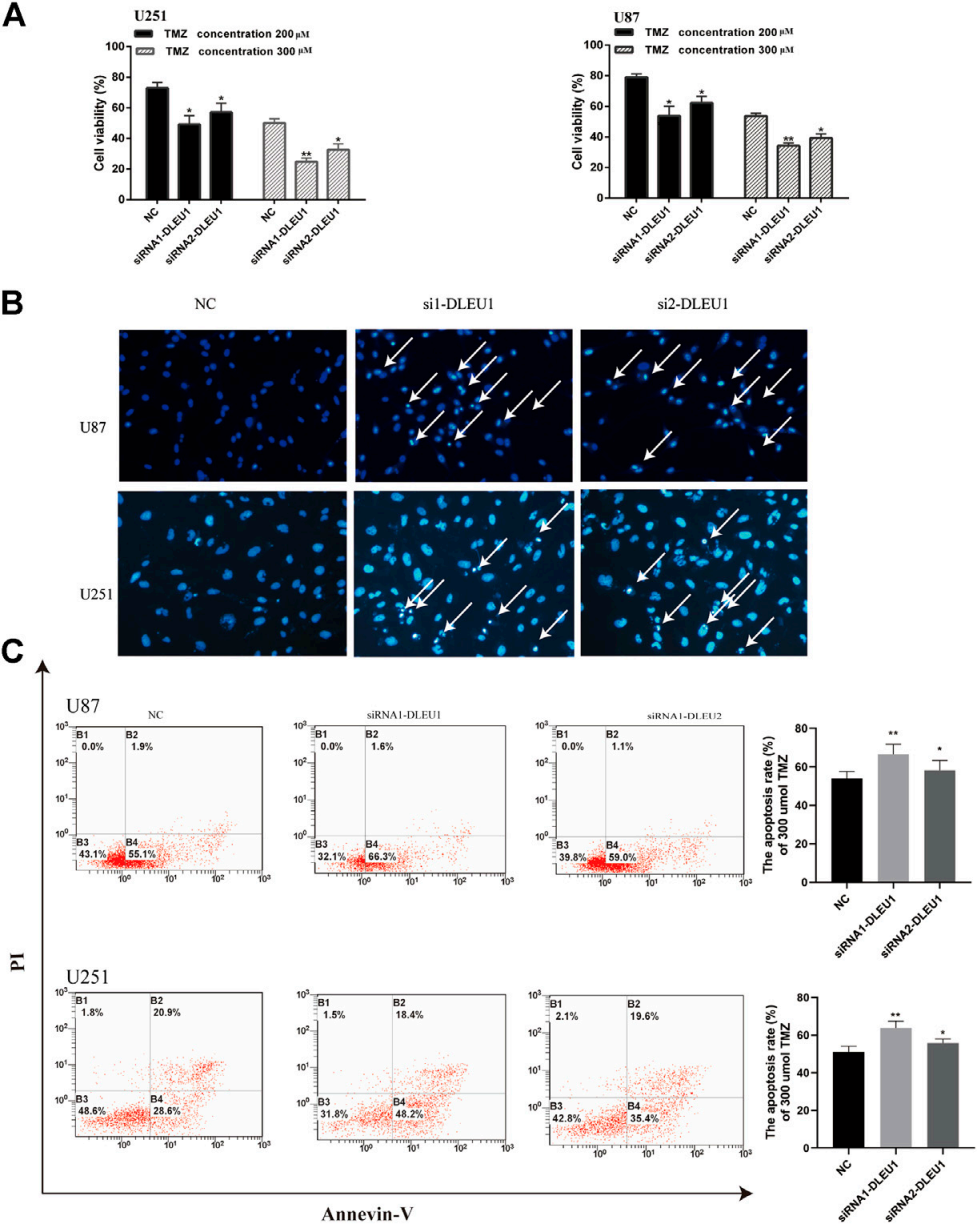
Knockdown of lncRNA DLEU1 contributed to TMZ sensitivity in glioma cells by promoting apoptosis. **(A)** MTS assay indicated that knock down of lncRNA DLEU1 increased TMZ cytotoxicity in glioma cells. **(B,C)** The apoptosis inU87 and U251 cells transfected with siRNA-DLEU1 was detected by Annexin-V and Hoechst 33342 staining, respectively. *p < 0.05, **p < 0.01, ***p < 0.001, NS, no significance.

Previous studies had shown that lncRNA DLEU1 was associated with drug resistance in bladder cancer on cisplatin (Li Y. et al., 2019). However, the role of lncRNA DLEU1 in TMZ sensitivity in glioma cells was unclear. Therefore, the effects of lncRNA DLEU1 on the drug sensitivity of U87 and U251 cells to TMZ were determined by MTS assay, discovering that transfection with lncRNA DLEU1 significantly improved the drug sensitivity of glioma cells to TMZ (Figure 6A). According to Figure 6B, Hoechst assay revealed that knocking down of lncRNA DLEU1 inhibited the cell viability and promoted apoptosis and the TMZ cytotoxicity in U87 and U251 cells at 72 h after transfection, compared with the NC group (300 μM TMZ).

Subsequently, we explored whether lncRNA DLEU1 affected TMZ sensitivity by facilitating apoptosis of glioma cells by Annexin V-FITC/PI staining. Flow cytometry analysis indicated that silencing of lncRNA DLEU1 accelerated the apoptosis of U251 and U87 cells treated with 300 μM TMZ for 48 h in comparison with NC groups (Figure 6C). Besides that, western bolt analysis was examined, showing that silencing of lncRNA DLUE1 could promote the expression of cleaved-PARP compared with NC group in U251 cells (SupplementaryFigure S1). All these findings revealed that knockdown of lncRNA DLEU1 could increase TMZ sensitivity in glioma cells by facilitating the apoptosis.

### Knock Down of lncRNA DLEU1 Can Enhanced Temozolomide Cytotoxicity in Glioma Cells by Inhibiting Temozolomide-Induced Autophagy

**FIGURE 7:**
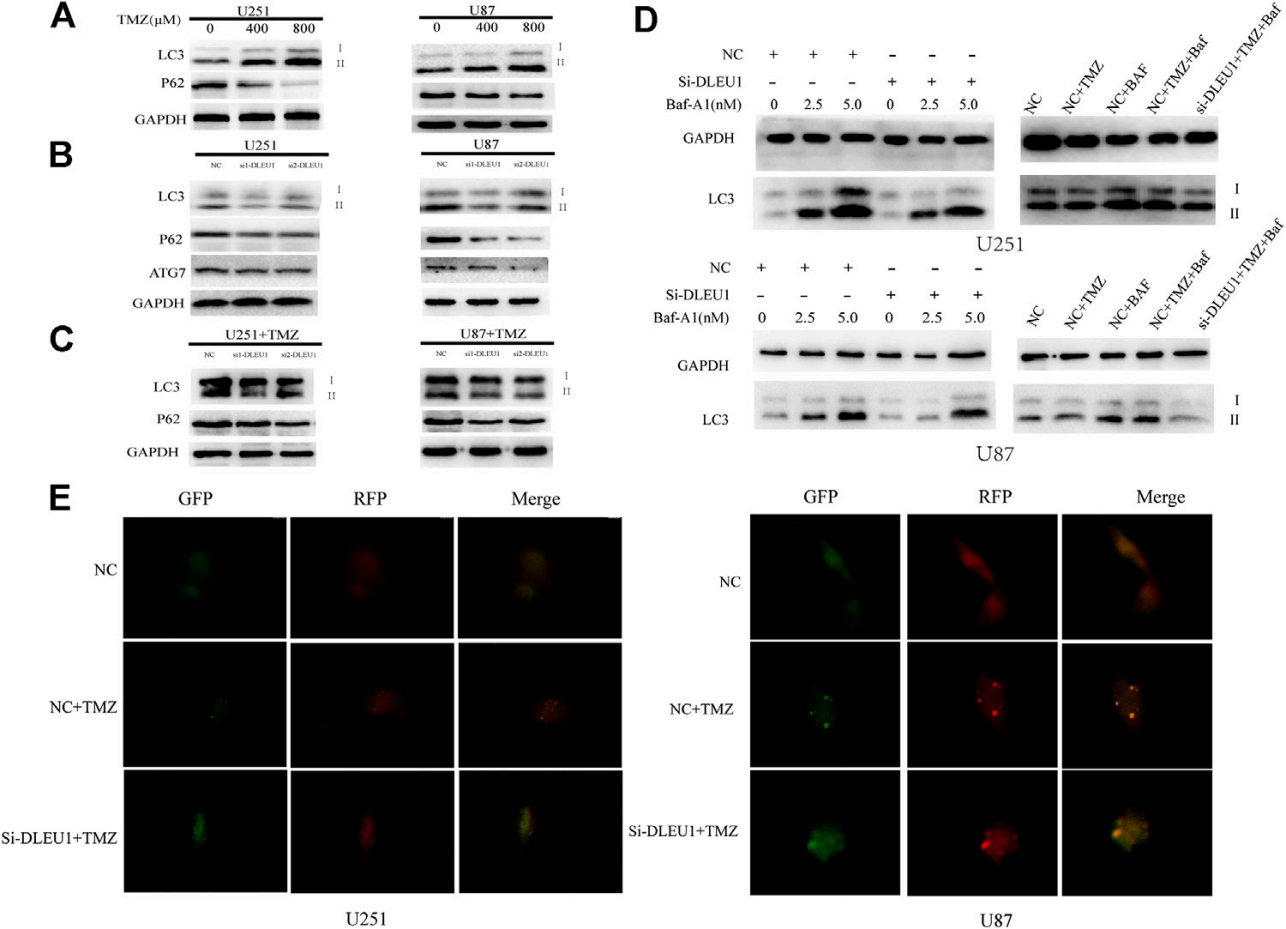
Knockdown of lncRNA DLEU1 inhibited the autophagy and might be involved in TMZ-induced autophagy. **(A)** Both transition from LC3I to LC3II and LC3-II expression were increased and P62 expression was decreased in glioma cells treated with different concentration TMZ. **(B)** Knockdown of lncRNA DLEU1 decreased the expression of transition from LC3I to LC3II, LC3-II, ATG7, and P62 in glioma cells. **(C)** TMZ inhibited the function of knockdown of lncRNA DLEU1 on transition from LC3I to LC3II, while P62 expression was still decreased. *p < 0.05, **p < 0.01, ***p < 0.001. **(D)** Knockdown of lncRNA DLEU1 increased the expression of LC3-II and the lC3II/I ratio in the presence of Baf-A1 and decreased the expression of LC3-II in the presence of BafA1 in glioma cells treated with TMZ. **(E)**. The number of yellow puncta was elevated in glioma cells treated with TMZ and knockdown of lncRNA DLEU1 treated with TMZ.

Previous studies had proved that TMZ-induced autophagy promoted the apoptotic of tumor cells, which contributed to treatment resistance (Li H. et al., 2018). To evaluate the effect of TMZ in activating autophagy, U87 and U251 cells were treated with different concentrations of TMZ (200, 400, 800 μM). Western blot analysis was carried out to examine the expression of autophagy markers such as LC3, ATG7, and P62. As shown in Figure 7A, we observed that the expression of P62 significantly diminished in a concentration-dependent manner, while the transition from LC3I to LC3II increased in U251 and U87 cells. Moreover, Figure 7B revealed that knockdown of lncRNA DLEU1 decreased protein expression of ATG7 and p62 in glioma cells, as well as the transition from LC3I to LC3II, compared with NC groups.

To make clear whether lncRNA DLEU1 could affect sensitivity of TMZ through autophagy, knockdown of lncRNA DLEU1 in U87 and U251 cells treated with 300 concentration of TMZ reduced the expression of P62 in glioma cells. However, transition from LC3I to LC3II in the siDLEU1 group had no significant change, compared with NC groups (Figure 7C). These results implied that knocking down lncRNA DLEU1 inhibited the autophagy and might be involved in TMZ-induced autophagy.

Bafilomycin A1(Baf-A1), an inhibitor of lysosomal degradation, could detect the change in autophagic flux by LC3II. Western blot analysis showed that knockdown of lncRNA DLEU1 decreased the protein expression of LC3-II in the presence of BafA1 in U251 and U87 cells (Figure 7D). Moreover, in Figure 7D, we also discovered that knockdown of lncRNA DLEU1 blocked the accumulation of LC3II in the presence of BafA1 in the glioma cells treated with TMZ. Meanwhile, the autophagy activating effect of TMZ and knockdown of lncRNA DLEU1 in glioma cells treated with TMZ were revealed by analysis of autophagic flux. Our results showed that lncRNA DLEU1 deletion could reduce the yellow puncta autophagosomes induced by TMZ in the absence of BafA1 (Figure 7E). All these results showed that knock down of lncRNA DLEU1 could enhanced TMZ cytotoxicity in glioma cells by inhibiting TMZ-induced autophagy.

**FIGURE 8:**
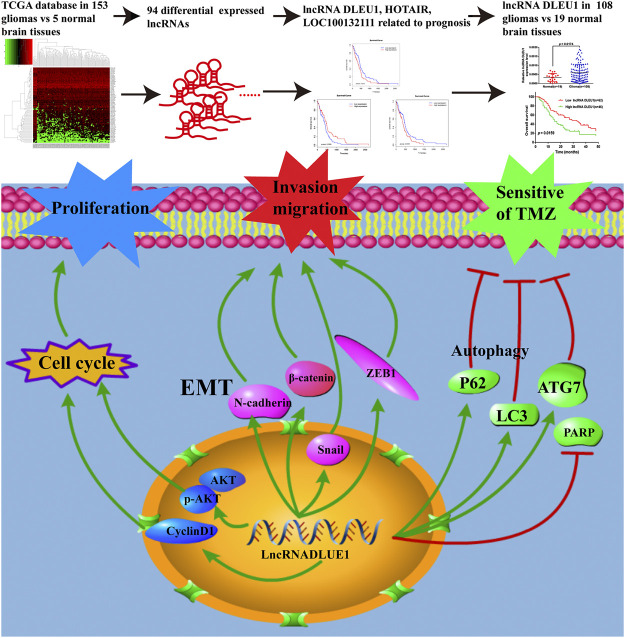
LncRNAs acted as biomarkers for diagnosis and prognosis of glioma and the underlying mechanisms of lncRNA DLEU1 for progression of glioma and TMZ chemosensitivity.

## Discussion

More and more potential molecular biomarkers have been explored and applied to enhance the diagnosis and treatment of gliomas. However, a dismal prognosis still remains (Xu et al., 2018). Thus, the therapeutic targets for glioma were always needed to be exploited. Recently, increasing reports had revealed that aberrant expression of lncRNAs could regulate the formation and development of tumors across numerous cancer types (Bartonicek et al., 2016). In this study, based on the TCGA database, we discovered 94 differentially expressed lncRNAs between gliomas and normal brain tissues. We identified 10 prognosis-related DEGs and predicted their underlying function. Additionally, lncRNA DLEU1, HOTAIR, and LOC00132111 were discovered to be significantly related to OS by using the log-rank test. Recent studies had demonstrated high-expressed lncRNA DLEU1 was significantly associated with advanced WHO grades, lower Karnofsky Performance Status (KPS) score, and proliferation of glioma cells (Feng et al., 2019; Wang J. et al., 2019). However, very few reports showed the prognosis value and molecular functions of abnormal lncRNA DLEU1 expression in gliomas.

We first detected the expression levels of lncRNA DLEU1 in 127 cases previously collected by another research group, discovering that the expression of lncRNA DLEU1 in glioma tissues was significantly higher than that in normal brain tissues. The Kaplan–Meier survival analysis and log-rank test were performed, showing that patients with a higher expression of lncRNA DLEU1 had a shorter overall survival rate, which was consistent with the results of TCGA database analysis. These results were compatible with the consequences found in colorectal cancer and gastric cancer (Li X. et al., 2018; Liu et al., 2018). To evaluate how lncRNA DLEU1 worked in glioma cells, we performed the MTS and colony formation assays, discovering that knockdown of lncRNA DLEU1 significantly suppressed the proliferation activity of glioma cells and decreased the number of newly formed colonies. Also, we demonstrated that lncRNA DLEU1 deletion induced cell cycle arrest at the G0/G1 phase and correspondingly decreased the percentage of S phase via regulating cell cycle transitions suppressed by *p*-AKT and cyclin D1. Concordantly with our results, knockdown of lncRNA DLEU1 suppressed the proliferation by downregulating AKT signal pathway in renal cell carcinoma (Yue et al., 2019).

Moreover, recent reports had demonstrated that knockdown of lncRNA DLEU1 significantly inhibited the invasion and migration abilities on osteosarcoma cells and glioma cells (Chen X. et al., 2019; Feng et al., 2019). Correspondingly, we found similar results that knockdown of lncRNA DLEU1 significantly inhibited the invasion and migration abilities not only in U87 cell but also in U251 cell. Moreover, the epithelial-mesenchymal transition (EMT) plays a crucial role in migration and invasion of various cancers (Lv et al., 2016b; Saitoh, 2018). Thus, we detected the protein levels of EMT-related markers, revealing that siRNA-DLEU1 suppressed the expression of EMT markers such as β-catenin, ZEB1, N-cadherin and snail. These observations mirrored lncRNA DLEU1 participate proliferation, invasion and migration of glioma cells by regulating the cell cycle and EMT.

Although a multitude of lncRNAs had been reported as crucial for drug resistance in various cancer (Abildgaard et al., 2019; Yang et al., 2020; Han et al., 2020), there were no studies evaluating the impact of drug resistance of lncRNA DLEU1. In the current study, we firstly detected the function of lncRNA DLEU1 on TMZ sensitivity by performing an MTS assay, Hoechst stain, and flow cytometry analysis, discovering that knockdown of lncRNA DLEU1 contributed to the TMZ sensitivity in U87 and U251 cells by enhancing the apoptosis. Concordantly with our results, Li et al. had provided the evidence that knockdown of lncRNA DLEU1 sensitized BCA cells to cisplatin-induced apoptosis (Li Y. et al., 2019). Recently, reports had been published that several biomarkers such as MGMT, STAT3, and APNG were involved in the TMZ resistance of gliomas (Jacinto and Esteller, 2007; Agnihotri et al., 2012; Lee et al., 2011). However, this drug resistance event appeared to be underrecognized. The interesting finding of our study that lncRNA DLEU1 could enhance the imply of TMZ on glioma cells provides a sensitizing strategy for TMZ treatment.

Several studies have demonstrated that autophagy that could be induced by anticancer therapies acted as a novel mechanism of chemoresistance by modifying P62 and LC3 and promoted cancer cell death (Lee et al., 2015; Zanotto-Filho et al., 2015; Yoshida, 2017). In our study, the role of lncRNA DLEU1 in autophagy was first explored in glioma cells. Western blot analysis showed that knock down of lncRNA DLEU1 dramatically reduced the LC3II expression, transition from LC3I to LC3II, as well as the expression of p62 and ATG7, which suggested that lncRNA DLEU1 deletion could suppress autophagy in glioma cells. Moreover, we evaluated the expression of LC3 and P62 treated under the increasing concentration of TMZ. In addition, we observed that TMZ increased the transition from LC3I to LC3II, while decreasing the protein expression of P62, which implied that the autophagy effect was activated by TMZ in glioma cells. Similar results that the autophagy activating effect of TMZ in glioma cells was previously confirmed (Lee et al., 2015; Zanotto-Filho et al., 2015; Wen et al., 2019). We also discovered that knockdown of lncRNA DLEU1 treated with TMZ could reduce the effect of lncRNA DLEU1 on autophagy. These results indicated that lncRNA DLEU1 might be involved in TMZ-induced autophagy and influenced the sensitivity of TMZ by enhancing the apoptosis that could be delayed by TMZ-induced autophagy (Lin et al., 2012; Rosenfeld et al., 2010; Hu et al., 2012). In order to evaluate the relation among autophagy, lncRNA DLEU1 and TMZ, autophagy flux assay and western bolt were performed, and the results showed that TMZ could induced the autophagy, and knockdown of lncRNA DLEU1 could reduce autophagosomes induced by TMZ and the protein expression of LC3II. In Figure 6A, we discovered that knockdown of lncRNA DLEU1 increased TMZ cytotoxicity in glioma cells. These results revealed knock down of lncRNA DLEU1 could enhanced TMZ cytotoxicity in glioma cells by inhibiting TMZ-induced autophagy.

In our study, we observed for the first time that lncRNA DLEU1 predicted a worse prognosis and might be involved in TMZ-induced autophagy, and deletion of lncRNA DLEU1 dramatically increased the sensitivity of U87 and U251 cells to TMZ. However, these findings are required to be further validated *in vivo*. Given that lncRNA DLEU1 expression was significantly up-regulated in glioma samples, a therapy targeting lncRNA DLEU1 could provide a prospective strategy for glioma treatment.

## Conclusion

In summary, as shown in Figure 8, on account of the TCGA database and collected glioma specimens, lncRNA DLEU1 was high expressed in gliomas and could serve as an independent prognostic biomarker for glioma patients. In addition, knockdown of lncRNA DLEU1 suppressed glioma progression by inducing cell cycle arrest, and inhibited the invasion and migration by depressing EMT. Furthermore, lncRNA DLEU1 deletion could promote TMZ sensitivity to glioma cells via enhancing apoptosis and regulating autophagy, which enriched the mechanism of temozolomide sensitivity and provided a potential biomarker for the individualized use of temozolomide in glioma.

## Data Availability Statement

The datasets presented in this study can be found in online repositories. The names of the repository/repositories and accession number(s) can be found in the article/Supplementary Material.

## Ethics Statement

The studies involving human participants were reviewed and approved by (No. 201803806). The patients/participants provided their written informed consent to participate in this study.

## Author Contributions

S-hC planned and supervised the study. Q-LL and L-cW contributed to the conception and design, data capturing and manuscript drafting, and performed in vitro experiments. D-CL, Y-LJ, ML, Q-XL, C-ZQ, and H-YL are responsible for collecting tissues and clinical information. X-LS drafted the article or critically revised it for important intellectual content. All authors read and agreed to the final manuscript.

## Funding

This work was supported by the National Natural Science Foundation of China (No. 81860664, 81703780, 81960458, 82060680), the Province Natural Science Foundation of Jiangxi Province (NO. 20192BAB215063, 20202BABL216080), Science and Technology Plan of Health and Family Planning Commission of Jiangxi Province (NO.20191091, 20195424), Traditional Chinese Medicine Science Research Fund of Jiangxi Province (NO. 2019A024, 2019A322), Science and Technology Research Project of Jiangxi Provincial Department of Education (180077).

## Conflict of Interest

The authors declare that the research was conducted in the absence of any commercial or financial relationships that could be construed as a potential conflict of interest.
