# Supplementary material for: Knockdown lncRNA DLEU1 Inhibits Gliomas Progression and Promotes Temozolomide Chemosensitivity by Regulating Autophagy
Source: Front Pharmacol. 2020 Dec 9;11:560543. doi: 10.3389/fphar.2020.560543 (PMC7756250; doi:10.3389/fphar.2020.560543)
Supplement: Supplementary file 1 [file table1.docx]

**Knockdown lncRNA DLEU1 inhibits gliomas progression by inhibiting** **EMT and promotes temozolomide** **chemosensitivity by regulating autophagy**

**Qiao-li Lv^1^**†**, Li-chong Wang^1,2,^**†**, Dang-chi Li^3^, Qian-xia Lin^4^, Xiao-li Shen^2^, Hai-yun Liu^4^, Min Li^5^, Yu-long Ji^4^, Chong-zhen Qin^6^, Shu-hui Chen^7^***


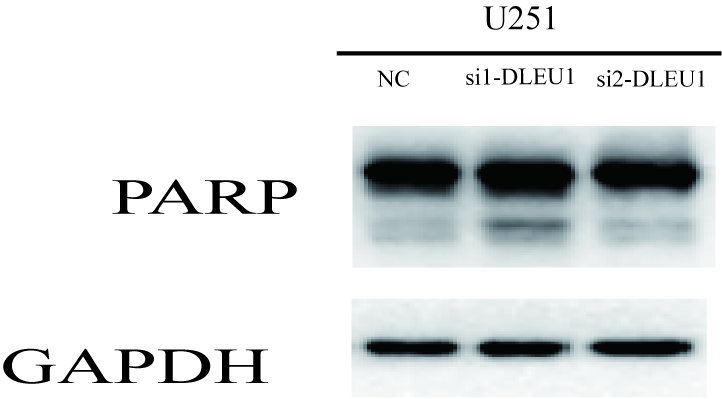


Figure1s the changes of PARP in protein levels.
